# Paediatric admissions to an adult ICU in a district general hospital in the UK

**DOI:** 10.1186/cc9908

**Published:** 2011-03-11

**Authors:** A Hazara, V Singh, P Davoudian

**Affiliations:** 1 Addenbrooke's NHS Foundation Trust, Cambridge, UK

## Introduction

Data were collected for paediatric admissions to an adult ICU in a district general hospital in the UK over a 6-year period to look for feasibility and the associated problems of training and skills after centralisation of PICUs in the country.

## Methods

We searched the ICNARC database for all entries relating to children for their age, gender, and diagnosis at admission, source of referral, length of stay, level of support, outcome/discharge and any other problems highlighted during their stay in the unit between the years 2002 and 2009.

## Results

Thirty-eight children were admitted to the adult critical care unit during this period. The age range was from 6 months to 16 years (average 9.12 years). Seventeen patients were male and 23 female. The most common reason for admission to the unit was respiratory problems followed by trauma. Seventeen patients received level 2 care and 21 received level 3 care. Twenty children needed endotracheal intubations, 12 needed arterial lines, 10 needed central lines and two needed intercostal drains. Twenty-one patients received sedation, most commonly with midazolam and morphine. The average length of stay in the ITU was 1.5 days and 80% of patients were discharged from the ITU within 2 days. Twenty-two patients were discharged to the ward Data are in total numbers (*n*) or proportions (%). *P < 0.05, **P < 0.01 for comparisons between two groups. ^#^Relative risk = 1.364 (95% CI: 1.055 to 1.763) compared with Group 2 (protocol). and 16 were transferred to a specialist ICU. Thirteen of these specialist transfers were to a local specialist centre and three to other specialist centres. The national paediatric transport service was used in seven instances, and local service in nine instances. The number of admissions to the ICU was few, and it was able to manage the cases and institute appropriate therapy. Less than 50% of these patients were transferred to a speciality hospital and most level 2 care could be managed in the district general hospital. In those needing transfer to specialist units, the availability of protocols for sedation and analgesia resulted in less delay in handover and transfers. Communications between various teams involved in transfer and preparation was effective and no critical incidents were reported.

## Conclusions

With the specialist centre bed occupancy remaining high, district general ICUs provide more and more ongoing level 2 care to critically ill children. This also confirmed the findings of other studies that widespread use of a specialist retrieval service has not resulted in loss of vital stabilisation skills.
